# Difference in clinical features of SARS-CoV-2 in pediatric patients before and after emergence of P.1

**DOI:** 10.1038/s41390-022-02046-3

**Published:** 2022-04-13

**Authors:** Char Leung, Ka-Wah Khong, Kwok-Hung Chan, Marcus Vinicius Guimarães Lacerda, Carlos Henrique Michiles Frank

**Affiliations:** 1 School of Clinical Medicine, University of Cambridge, Cambridge, Cambridgeshire UK; 2 Deakin University, Burwood, VIC Australia; 3 Faculty of Medicine, University of Hong Kong, Pokfulam, Hong Kong; 4 State Key Laboratory for Emerging Infectious Diseases, Department of Microbiology, Queen Mary Hospital, University of Hong Kong, Pokfulam, Hong Kong; 5 Fundação de Medicina Tropical Dr Heitor Vieira Dourado, Manaus, Amazonas Brazil; 6 Instituto Leônidas & Maria Deane, Fundação Oswaldo Cruz, Manaus, Amazonas Brazil

## Abstract

**Background:**

The P.1 variant is a Variant of Concern announced by the WHO. The present work aimed to characterize the clinical features of pediatric patients with SARS-CoV-2 before and after the emergence of P.1.

**Methods:**

This is a cohort study. Data of symptomatic patients younger than 18 years diagnosed with COVID-19 by PCR tests registered in *Painel COVID-19 Amazonas* were analyzed.

**Results:**

A total of 4080 symptomatic pediatric patients were identified in the database between March 2020 and July 2021, of which 1654 were categorized as pre-P.1 and 978 as P.1-dominant cases, based on the prevalence of P.1 of >90% in the North Region, Brazil. Lower case-fatality rate was observed in non-infants infected during the P.1-dominant period (0.9% vs. 2.2%). In general, patients infected during the P.1-dominant period had less fever (70.8% vs. 74.2%) and less lower respiratory tract symptoms (respiratory distress: 11.8% vs. 18.9%, dyspnea: 27.9% vs. 34.5%) yet higher prevalence of neurological symptoms, headache for example (42.8% vs. 5.9%).

**Conclusions:**

The prevalence of symptoms of COVID-19 can differ across different periods of variant dominance. Lower prevalence of fever during the P.1-dominant period may reduce the effectiveness of symptom-based screening in public premises where laboratory diagnostic tests are not available.

**Impact:**

- The prevalence rate of symptoms of SARS-CoV-2 infection can differ among different variants.
- The present work documents the difference in the clinical features of SARS-CoV-2 in patients aged below 18 years before and after the emergence of P.1, the first study of its kind.
- Unlike previous studies that focus solely on hospitalized cases, the present work considers both mild and severe cases.
- While non-infants had a lower fatality rate, lower prevalence of fever associated with the emergence of P.1 may reduce the effectiveness of symptom-based screening in public premises where laboratory diagnostic tests are not available.

## Introduction

Originating in Wuhan,China in 2019, SARS-CoV-2 has resulted in the current COVID-19 pandemic that remains a global health concern despite measures of infection control. In response, the World Health Organization has categorized these variants into “variants under monitoring”, “variants of interest”, and “variants of concern (VOC)”, depending on their impacts on global public health significance.^1^ The latter poses a greater concern, signifying increased transmissibility, a change in clinical presentations, or a decrease in effectiveness of public health measures, vaccines, or therapeutics. As of 19th December, 2021, five variants had been classified as variants of concern, including Alpha first reported in the UK, Beta in South Africa, Gamma in Brazil, Delta in India, and Omicron in South Africa.

**Fig. 1:**
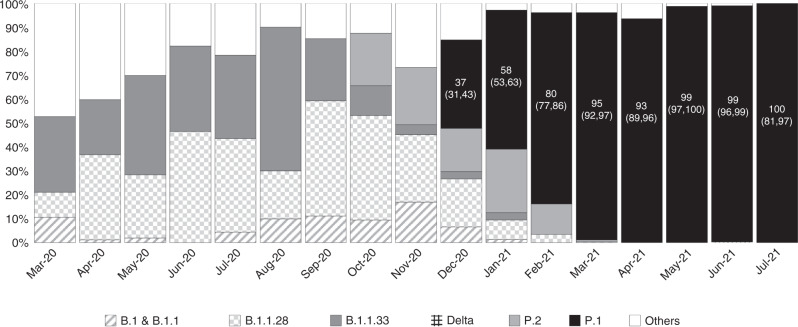
Lineage prevalence of P.1 in the North Region of Brazil, % (95% CI).

Characterized by three mutations in the spike protein receptor binding domain K417T, E484K, and N501Y^2^, the Gamma variant, also called the P.1 lineage, is one of the two major variants originating in Brazil, along with the Zeta variant P.2 first detected in Rio de Janeiro. It was first identified in travelers coming from Amazonas, Brazil to Tokyo in January 2021. Based on samples collected in Manaus, the capital of the state of Amazonas and the most populated city in the North Region, a study by Faria et al.^3^ suggested that P.1 emerged in Brazil in November 2020. They detected P.1 in 85% of the samples collected between January 1 and 8, 2021, resulting in an estimated lineage prevalence (defined as the percentage of individuals in a population who are infected with the lineage) of almost 90% by February 2021.^3,4^ According to the Oswaldo Cruz Foundation (*Fiocruz*)^5^, the lineage prevalence of P.1 in the North Region remained above 90% from March to July 2021 (Fig. 1). Consequently, it has been suggested that P.1 is 2.6 times more transmissible than previous circulating variants.^6^

In addition to increased transmissibility, the effectiveness of vaccines and treatment is another concern. Significantly reduced vaccine-induced antibody neutralization of P.1 was observed in the sera of subjects administered with BNT162b2, mRNA-1273 and Ad26.COV2.S vaccine, compared with the wild-type and other variants.^7–9^ Moreover, P.1 was found to be more resistant to neutralization by convalescent plasma.^9^ The emergency use of bamlanivimab and etesevimab administered together was previously approved by the Food and Drug Administration for the treatment of mild to moderate SARS-CoV-2 infection in adults and pediatric patients. However, results from in vitro assays suggested that the combined use of these monoclonal antibodies was not active against P.1. As a result, the distribution of these monoclonal antibodies was paused by the US Government.^10^

Despite these concerns, little is known about the clinical presentations of P.1^11^, hindering early screening and diagnosis in primary care settings. Based on a systematic review of literature on P.1 in PubMed, Embase, and Lilacs (Latin American and Caribbean Health Sciences Literature) (see Supplementary Materials S.1), only 8 studies comprising 11 cases described the clinical features of P.1 and none of these cases involved the pediatric population. Against this background, the present work aims to clarify the difference in clinical features between pediatric patients during the pre-P.1 and P.1-dominant period, the first study of its kind.

## Methods

### Study design and participants

The clinical and demographic data of all confirmed cases of COVID-19 in the Amazonas state were collected on the 2nd August 2021 from Amazonas COVID-19 Panel (*Painel COVID-19 Amazonas*), a statewide database of COVID-19 cases managed by Amazonas State Health Secretariat (*Secretaria de Estado de Saúde do Amazonas*) as part of the state government’s commitment to the transparency of information. Reporting of cases of Compulsory Reporting Diseases (*Doenças de Notificação Compulsória, DNC*), such as COVID-19, tuberculosis, leprosy, and measles, is mandatory in Amazonas. The data registered in the database were collected by Amazonas State Health Secretariat information systems of municipal health departments (*Secretarias Municipais de Saúde*). Details of the database such as sources of data can be found in Supplementary Materials (S.2). Basic demographic, epidemiologic and medical data such as sex, age, ethnicity, location, vaccination against SARS-CoV-2, diagnostic method of SARS-CoV-2 infection (RT-PCR test and rapid test), clinical status as of data collection (recovered, died of COVID-19, and died of other causes), signs and symptoms, and comorbidities were systematically registered whereas information such as therapeutics and laboratory results were not available.

Data of all COVID-19 cases confirmed between 13th March 2020 (the first COVID-19 case in Amazonas) and 31st July 2021 were gathered from the database. With the lineage prevalence of P.1 reaching nearly 100% in the North Region in March 2021 as discussed, cases confirmed between 13th March and 31st October 2020 were considered pre-P.1 whereas those confirmed between 1st March and 31st July 2021 were considered P.1-dominant. In addition, asymptomatic cases were excluded to reduce selection bias because only symptomatic patients were eligible for SARS-CoV-2 diagnostic tests in Amazonas in 2020. Therefore, cases meeting all following criteria were included in the statistical analyses in comparing P.1-dominant and pre-P.1 group: (i) SARS-CoV-2 infection was diagnosed with RT-PCR tests, (ii) patients aged below 18 years, (iii) symptomatic, and (iv) cases confirmed during the above-mentioned time periods; namely, March to October 2020 and March to July 2021. The following data were extracted for analysis, (i) age, (ii) sex, (iii) COVID-19-related death, (iv) ethnicity, (v) location by municipality, (vi) symptoms, and (vii) preexisting comorbidities. With the exception of age, all other variables are dichotomous. Age groups were also constructed as dichotomous variables according to following definitions, infants (age 0–1 year), young children (2–6 years), children (7–12 years), and adolescents (13–17 years). Ethnicity was self-identified consisting of categories Hispanic (*preta/parda*), Asian (*amarela*), Caucasian (*branca*), and indigenous (*indígena*). Location referred to the municipality at which the case was notified. For simplicity, municipalities were grouped into four intermediate geographic regions (*região geográfica intermediária*)—Manaus, Parintins, Tefé, and Lábrea - according to the Brazilian Institute of Geography and Statistics (*Instituto Brasileiro de Geografia e Estatística, IBGE*). Details can be found in Supplementary Materials (S.3). Signs and symptoms include ageusia, anosmia, coryza, cough, diarrhea, dyspnea, fatigue, fever, headache, respiratory distress, sore throat, vomiting, and others. Comorbidities include heart disease, hematologic disease, neurologic disease, liver disease, renal disease, immunosuppression, Down’s syndrome, obesity, diabetes, and others. The number of comorbidities was dichotomized and categorized into four levels as none, one, two, and three or more.

Data used in the present analysis were collected from the publicly available database and are already de-identified, therefore ethical approvals in Brazil and Hong Kong are not required.

### Outcomes

Given the increased transmissibility and concerns about vaccine and therapeutics effectiveness of the P.1 lineage, the primary outcome was the difference in clinical features between Pre-P.aand P.1-dominant cases in the underage. Factors including age, ethnicity, and preexisting conditions were taken into consideration as potential confounders in identifying clinical features that were more likely to be associated with the dominance of P.1.

### Statistical analysis

The data of cases confirmed between 13th March 2020 and 31st July 2021 were first analyzed in the form of (i) weekly number of confirmed cases, and (ii) correlation between the monthly prevalence rate of symptoms and the monthly lineage prevalence of each of the following strain, (i) B.1 and B.1.1, (ii) B.1.1.28, (iii) B.1.1.33, (iv) B.1.617.2 (also known as the Delta strain), (v) P.2, (vi) P.1, and (vii) others that aim to examine the impact of different variants on signs and symptoms.

Next, statistical comparisons of the baseline characteristics between P.1-dominant and pre-P.1 cases were made in three ways, (i) Fisher’s exact tests for comparing two proportions were used for dichotomous variables (the Chi-square test is rather an approximate test to Fisher’s exact test) whereas *t* tests or Mann–Whitney tests were used for median age or mean age, depending on the data normality condition; (ii) a histogram was used to assess the difference in distribution of age between the two groups; and (iii) The multivariate logistic regression was used to identify independent factors associated with P.1-dominant cases, taking into account possible confounding effects among these measures. Infants and Manaus were used as the reference group for age group and location variables, respectively. The forward stepwise procedure was adopted for variable selection with *p* value < 10% as the rule for variable inclusion. The overall accuracy of the regression was assessed by the area under the receiver operating curve characteristic (ROC) curve. To assess the robustness of the multivariate analysis, a sensitivity analysis was conducted by repeating the multivariate analysis using a subset of the data consisting of only cases of children and adolescents.

Complete data were not available for all variables. For any missing data on signs, symptoms, or comorbidities, the clinical condition is assumed to be absent. For ethnicity, cases with missing data were removed from statistical analyses that involve ethnicity. All statistical analyses were carried out using statistical software R version 3.6.1. A *p* value < 0.05 was considered significant.

## Results

**Fig. 2:**
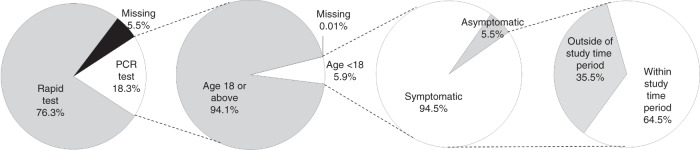
Case inclusion procedure (2nd July 2021).

As of 2nd August 2021, a total of 404,262 cases dated between 16th March 2020 and 31st July 2021 were identified in the database, of which 210,929 (52.2%) were confirmed in 2020. After the removal of 330,389 cases using rapid tests for diagnosis (76.2%) and missing data (5.5%), 73,873 cases (18.3%) with PCR tests for diagnosis were left for consideration (Fig. 2). Of these, 4,318 patients aged below 18 years (5.8%) and the remaining cases consisted of 69,420 patients aged ≥18 years (94.0%) and 10 cases with no age specified (0.01%). Of these 4318 patients, 4080 (94.5%) were symptomatic. A detailed breakdown of all cases identified in the database can be found in Supplementary Materials (S.4).

The weekly average of confirmed cases was 55.8 with a standard deviation of 32.6. Of the 4080 cases, 2632 cases (64.5%), were confirmed within the study time period and hence included in statistical analysis, including 1,654 confirmed in the pre-P.1 timeframe (March–October 2020) and 978 in the P.1-dominant timeframe (March–July 2021). Of the included cases, seven patients died of non-COVID-19-related causes (0.3%) and 99 cases had ethnicity status missing (3.8%).

**Fig. 3:**
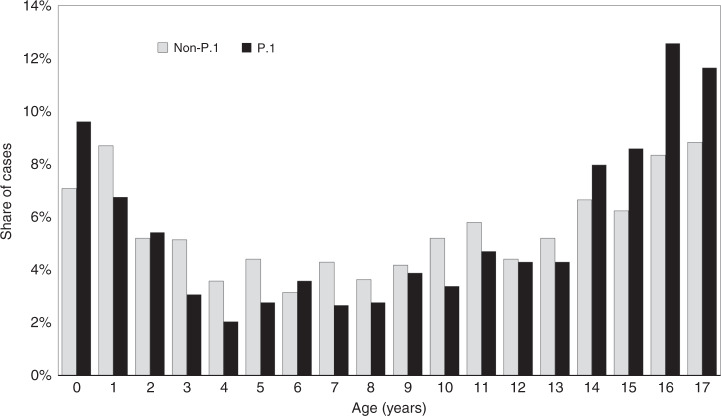
Share of symptomatic cases by age and strain.

The median age of P.1-dominant and pre-P.1 was 11 and 10 years, respectively (Table 1) and the difference was significant (*p* < 0.001). The age distribution of the two groups is shown in Fig. 3. It appears that those aged above 13 years were more affected during the P.1-dominant period. Similar findings can be concluded from Table 1 with adolescents being more affected during this period (45.1% vs. 35.2%; *p* < 0.001) while children (21.7% vs. 27.5%; *p* = 0.001) and young children (16.9% vs. 21.5%; *p* = 0.005) were less affected. Infants were equally affected in both periods (16.4% vs. 15.8%; *p* = 0.700). The case-fatality rate (CFR) of the P.1-dominant and pre-P.1 cohort was 1.1% and 2.1% (*p* = 0.065). However, significant difference in the CFR was observed in non-infants (*p* = 0.017) with lower CFR in the P.1-dominant group (0.9% vs. 2.1%).

**Table 1:** Demographic and baseline statistics of the studied cohort.

	P.1 (*n* = 978)	Non-P.1 (*n* = 1654)	*p* value (P.1 vs. non-P.1)
Age, y, median	11.00 (978)	10.00 (1654)	<0.001
Male, %	44.17 (432/978)	49.82 (824/1654)	0.005
Age groups, %			
Infants	16.36 (160/978)	15.78 (261/1654)	0.700
Young children	16.87 (165/978)	21.46 (355/1654)	0.005
Children	21.68 (212/978)	27.51 (455/1654)	0.001
Adolescents	45.09 (441/978)	35.25 (583/1654)	<0.001
Case-fatality rate, %			
All	1.12 (11/978)	2.12 (35/1654)	0.065
Non-infants	0.86 (7/818)	2.23 (31/1393)	0.017
Infants	2.50 (4/160)	1.53 (4/261)	0.486
Young children	0.61 (1/165)	2.82 (10/355)	0.187
Children	0.94 (2/212)	1.98 (9/455)	0.517
Adolescents	0.91 (4/441)	2.06 (12/583)	0.203
Ethnicity, %			
Latino	87.15 (807/926)	82.45 (1325/1607)	0.002
Asian	0.86 (8/926)	1.87 (30/1607)	0.060
Caucasian	8.96 (83/926)	7.90 (127/1607)	0.369
Indigenous	3.02 (28/926)	5.60 (90/1607)	0.003
Location, %			
Manaus	75.77 (741/978)	77.93 (1289/1654)	0.212
Parintins	3.68 (36/978)	2.11 (35/1654)	0.018
Tefé	9.92 (97/978)	12.45 (206/1654)	0.050
Lábrea	10.63 (104/978)	7.50 (124/1654)	0.006
Signs and symptoms, %			
Ageusia	15.03 (147/978)	3.51 (58/1654)	<0.001
Anosmia	15.44 (151/978)	11.00 (182/1654)	0.001
Coryza	25.15 (246/978)	3.69 (61/1654)	<0.001
Cough	61.86 (605/978)	66.08 (1093/1654)	0.032
Diarrhea	2.97 (29/978)	8.83 (146/1654)	<0.001
Dyspnea	27.91 (273/978)	34.52 (571/1654)	<0.001
Fatigue	5.32 (52/978)	2.30 (38/1654)	<0.001
Fever	70.76 (692/978)	74.18 (1227/1654)	0.057
Headache	42.84 (419/978)	5.86 (97/1654)	<0.001
Respiratory distress	11.76 (115/978)	18.92 (313/1654)	<0.001
Sore throat	38.24 (374/978)	29.32 (485/1654)	<0.001
Vomit	3.68 (36/978)	8.46 (140/1654)	<0.001
Others	14.52 (142/978)	17.29 (286/1654)	0.063
Comorbidities, %			
No comorbidities	95.19 (931/978)	92.44 (1529/1654)	0.005
1 comorbidity	3.48 (34/978)	5.26 (87/1654)	0.034
2 comorbidities	1.33 (13/978)	1.75 (29/1654)	0.427
≥3 comorbidities	0.00 (0/978)	0.54 (9/1654)	0.031
Heart disease	0.92 (9/978)	0.73 (12/1654)	0.652
Hematologic disease	0.00 (0/978)	0.73 (12/1654)	0.005
Neurological disease	0.61 (6/978)	1.21 (20/1654)	0.157
Hepatic disease	0.00 (0/978)	0.12 (2/1654)	0.533
Renal disease	0.31 (3/978)	0.42 (7/1654)	0.753
Immunodeficiency	0.92 (9/978)	1.45 (24/1654)	0.279
Down syndrome	0.51 (5/978)	0.48 (8/1654)	>0.999
Obesity	0.41 (4/978)	0.18 (3/1654)	0.435
Diabetes	0.10 (1/978)	0.48 (8/1654)	0.167
Others	2.35 (23/978)	4.66 (77/1654)	0.003

For signs and symptoms, fever (70.8%) and cough (61.9%) remained the most prevalent symptoms during the P.1-dominant period. However, their prevalence rates were lower in the P.1-dominant group compared with the pre-P.1 group (fever 74.2% and cough 66.1%). Lower respiratory tract symptoms reported by the patients also had lower prevalence rates in the P.1-dominant cohort—about 27.9% of the patients had dyspnea, compared with 34.5% in the pre-P.1 cohort and the difference was significant (*p* < 0.001). Similarly, respiratory distress was observed in 11.8% of the patients infected during the P.1-dominant period but was observed in 18.9% of the patients in the pre-P.1 period, and again the difference was significant (*p* < 0.001). In contrast, patients in the P.1-dominant group had more upper respiratory tract symptoms such as coryza (25.2% vs. 3.7%; *p* < 0.001) and sore throat (38.2% vs. 29.3%; *p* < 0.001). In addition, neurological symptoms were more common in the P.1-dominant cohort, seeing higher prevalence rates of ageusia (15.0% vs. 3.5%; *p* < 0.001) and anosmia (15.4% vs. 11.0%; *p* = 0.001). Furthermore, patients infected during the P.1-dominant period had seven-fold higher prevalence of headache (42.8% vs. 5.9%) than non-P.1.

As demonstrated in the results of the multivariate analysis (Table 2), only a number of clinical features were independently associated with the P.1-dominant group. After taking into account the confounding effects such as sex, age groups, and ethnicity, four signs and symptoms were found independently associated with the dominance of P.1, including coryza (MOR 4.378 [95% CI 3.08–6.22]; *p* < 0.001) and two neurological symptoms, ageusia (MOR 2.37 [95% CI 1.61–3.49]; *p* = 0.001) and headache (MOR 9.56 [95% CI 7.30–12.52]; *p* < 0.001). There is some evidence suggesting the independent role ethnicity plays in the dominance of P.1 infection, as indicated by the Hispanic group (MOR 1.43 [1.08–1.89]; *p* = 0.012). Because preexisting conditions are not common in pediatric patients, almost none of the comorbidities were found associated with the P.1-dominant group, with the exception of hematologic disease, albeit not significant (multivariate OR [MOR] < 0.001 [95% CI 0–Inf]; *p* = 0.956). The complete results of the univariate analysis can be found in Supplementary Materials (S.5).

**Table 2:** Results of multivariate logistics regression with 95% confidence intervals.

	Multivariate ORs	*p* value
Male	0.78 (0.65, 0.95)	0.013
Children	0.72 (0.58, 0.91)	0.005
Latino^a^	1.43 (1.08, 1.89)	0.012
Ageusia	2.37 (1.60, 3.49)	<0.001
Coryza	4.38 (3.08, 6.22)	<0.001
Diarrhea	0.41 (0.26, 0.65)	<0.001
Fatigue	4.42 (2.70, 7.23)	<0.001
Fever	0.72 (0.58, 0.89)	0.003
Headache	9.56 (7.30, 12.52)	<0.001
Other symptoms	0.63 (0.48, 0.84)	0.001
Hematologic disease	0.00 (0.00, Inf)	0.956
Parintins^b^	3.23 (1.94, 5.38)	<0.001
Lábrea^b^	1.44 (1.03, 2.01)	0.034

Results adjusted for sex, age, ethnicity, signs and symptoms, comorbidities, and locations.

^a^Caucasian.

^b^Manaus was used as the reference group.

For the multivariate analysis, the area under the receiver operating characteristic (ROC) curve was 0.79 (95% CI 0.77–0.81), indicative of excellent accuracy. The graphical illustration of the ROC curve can be found in Supplementary Materials (S.6).

The multivariate analysis is robust as demonstrated by the similar results between the sensitivity analysis and the original analysis that was based on the entire dataset. The MORs in the sensitivity analysis were similar to their counterparts in the original analysis, for instance Hispanic (95% CI 1.08–1.89 in the original analysis vs. 1.33–2.78 in the sensitivity analysis), ageusia (95% CI 1.61–3.49 vs. 1.36–3.37), and fever (95% CI 0.58–0.89 vs. 0.53–0.90). Complete results can be found in Supplementary Materials (S.7).

Looking beyond the comparison between P.1-dominant and pre-P.1 period, the monthly prevalence rates (including those not in the study period, i.e., November 2020 to February 2021) of some symptoms were closely related to the monthly lineage prevalence of P.1. In line with our findings, the Pearson correlation coefficients between the lineage prevalence of P.1 and, coryza and headache are 0.88 and 0.91, respectively, indicative of strong association. Moderately positively correlation is observed in ageusia with coefficient of 0.72. Results concerning other signs and symptoms as well as other lineages can be found in Supplementary Materials (S.8).

## Discussion

Here, endeavor has been made to define clinical characteristics of different SARS-CoV-2 variants, such as transmissibility, severity, symptoms, age distribution, and so on. Although P.1 is believed to have higher transmissibility and severity, currently there is no published data on the characteristic symptoms and age distribution of cases.^12^ To our knowledge, the present work is the first study to describe clinical manifestations during the P.1-dominant period, providing important information for formulating a case definition used for population screening test. Major findings of the present work include lower CFR in non-infants infected during the P.1-dominant period. as well as less low respiratory symptoms and higher prevalence of neurological symptoms such as headache and ageusia in pediatric patients.

The CFR of non-infants infected during the P.1-dominant period have found to be lower compared with the pre-P.1 period, partly because of the reduced prevalence of lower respiratory tract symptoms. In a preprint by Freitas et al.^13^ that compared the risk of severity and fatality between November-December 2020 and February 2021 of COVID-19 patients in Rio Grande do Sul (South Region), no significant change in severity and CFR was found in those aged below 19 years. However, the lineage prevalence of P.1 in the South Region in February 2021 was reported to be 61%,^5^ meaning that a considerable number of COVID-19 cases were non-P.1. Furthermore, the study only considered hospitalized cases. In contrast, Funk et al.^14^ found a high risk for hospitalization and ICU admission in those aged below 60 years, based on a sample of 352 patients with P.1 of which 79 aged below 20 years. The difference in findings between the present work and existing literature may be attributed to the study design that the present work solely focuses on the pediatric population. Nevertheless, given the increased transmissibility of P.1, the finding of lower CFR tends to support the virulence-transmission trade-off hypothesis in which virulence is an unavoidable cost of with-in host replication and increasing this cost results in a deceleration in transmission rate because increasing with-in host replication increases mortality rates.^15^ If this hypothesis holds true for SARS-CoV-2, the disease severity of COVID-19 may decline in the future while more transmissible and future variants of SARS-CoV-2 may eventually evolve like coronaviruses 229E, HKU1, OC43, and NL63 that cause the common cold. Another explanation to the lower CFR observed might be that previous exposure to SARS-CoV-2 might lead to protection against the severe form of P.1 infection. Of note, the attack rate of SARS-CoV-2 in Manaus rose from 66 to 76% from June to October 2020 while that in São Paulo was 29% in October 2020,^16^ indicating a considerably high seroprevalence rate.

Noticeably, fever is still the most common sign but infections during the P.1-dominant period resulted in less fever. The use of fever as a selection criterion has been demonstrated to enhance the efficiency of screening.^17^ Given the lack of accurate diagnostic tests, symptom-based screening, including body temperature taking, has been popular in airports, schools and many public premises. In this regard, the finding of lower prevalence of fever in pediatric patients can potentially reduce the effectiveness of symptom-based screening in public premises. Transmission from children to household members was low owing to school closures.^18^ However, coupled with further loosened social distancing policies in the future, the relative lack of prominence of fever could magnify the role of children in SARS-CoV-2 transmission.

The present work also sheds some light on the susceptibility to P.1. Our analysis confirmed that adolescents are more vulnerable to SARS-CoV-2 infection compared with young children, irrespective of period of infection. This provides an explanation to the current observation of increased transmissibility in high school settings than in elementary school settings.^19^

Furthermore, because adolescents were found more affected during the P.1-dominant period, additional measures should be implemented in high schools located in P.1 prevalent areas. For ethnic groups, Hispanic appeared to be more susceptible during the P1.-dominant period. It is not clear this means that Hispanic was more vulnerable to P.1 than the Delta variant because the Delta variant only accounted for 3.7% of all VOC cases in Brazil as of end of July.^20^ If so, previous findings of ethnic disparity in SARS-CoV-2 infection may not only be attributed to sociodemographic factors but also genetic ones.

There are several possibilities contributing to the high prevalence of headache in patients infected during the P.1-dominant period. Many respiratory viruses can cause acute rhinosinusitis, so the headache reported could be facial pain associated with rhinosinusitis, known as sinus headache.^21^ However, when analyzing correlation between reported symptoms, both headache and coryza were identified as independent factors associated with the P.1-dominant group (Supplementary Materials S8), making this explanation less likely.

The major strength of the present work is the inclusion of mild and non-hospitalized cases. Existing studies^13,22^ concerning COVID-19 in Brazil analyzed data gathered from the database SIVEP-Gripe that only considered hospitalized cases. Moreover, the present work is unique in terms of the large sample size of pediatric patients. Nevertheless, the present study is not free from limitations. Only symptomatic cases were considered. However, the exclusion of asymptomatic cases can reduce selection bias attributed to the difference in diagnostic test criteria in Manaus in 2020, as addressed earlier. In addition, although an effort was made by the State Government of Amazonas to ensure data consistency, missing, inaccurate and biased data are generally inevitable owing to the nature of case registration in a point-of-care setting and symptoms that rely on self-reporting as well as disruption during the pandemic. Biases may also arise from factors the present work does not take into account. These factors include co-infection with influenza and vaccination against SARS-CoV-2. Having said that, the vaccination rate in the Brazilian pediatric population is very low with vaccination rate below 10% in the population below 18 years of age (assuming a pediatric population of 1.6 million according to IBGE and 140,000 individuals aged below 18 were vaccinated as of end of July 2021 [https://covid19.manaus.am.gov.br/transparencia-covid-19/]), and influenza and other respiratory virus activity remained low in Brazil during the studied period.^23^ Finally, while PCR tests were used to confirm SARS-CoV-2 infection, the data did not specify the type of strain of the infection. Nevertheless, cases confirmed during the P.1 period are very likely to be patients infected with P.1 given the very high lineage prevalence of nearly 100%.

## Supplementary information

Supplementary materials
