# Supplementary material for: Difference in clinical features of SARS-CoV-2 in pediatric patients before and after emergence of P.1
Source: Pediatr Res. 2022 Apr 13;93(1):176–82. doi: 10.1038/s41390-022-02046-3 (PMC9006205; doi:10.1038/s41390-022-02046-3)
Supplement: Supplementary file 1 — Supplementary materials [file 41390_2022_2046_MOESM1_ESM.pdf]

Supplementary materials

Supplementary material (S.1)

(a) Systematic review for literature on P.1

A search was performed in PubMed, Embase, and LILACS (Latin American & Caribbean Health Sciences Literature) on the 11th July 2021 with search terms, "SARS-CoV-2" AND ("Amazonas" OR "P.1 variant" OR "variant P.1" OR "P.1 strain" OR "strain P.1"). An article was considered if it contained information about the clinical features of P.1, such as that of individual cases and summary statistics. All types of studies were considered, including case reports, systematic reviews, editorials, commentary, etc. There was no restriction on language and year of publication. Articles with no mention of clinical features of P.1 were excluded. The following data were extracted: (i) location, (ii) age, (iii) sex, and (iv) signs and symptoms.

Identified articles were screened by two investigators (CL and MK) independently. In the screening process, duplicates were removed and, title and abstract analysis was performed to exclude articles not related to P.1. Full text assessment for eligibility was given to the remaining articles. Then articles describing the clinical features of P.1 were included and the clinical data were extracted.

The PRISMA flow diagram of the search results is shown below.

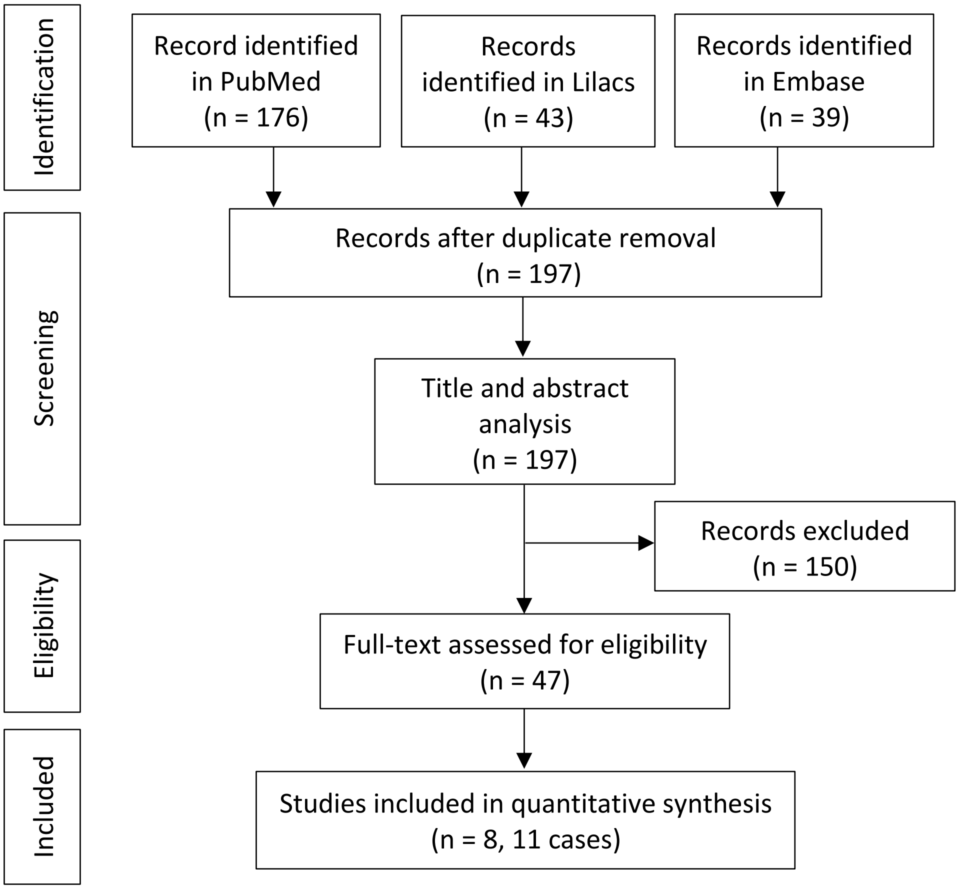

(b) Results of meta-analysis

Source	S1	S2	S3	S3	S4*	S5	S6	S7**	S8	S8	S8	
**Location**	Italy	Spain	Brazil	Brazil	Italy	Italy	Japan	Brazil	Brazil	Brazil	Brazil	
**Age**	83	44	36	64	22	33	46	26	69	41	71	Median: 44
**Male**	1	1	0	0	0	1	1	0	1	1	0	Male: 54.5%
**Signs and symptoms when SARS-CoV-2 detected**												Prevalence
**Ageusia**	0	0	0	0	0	0	0	0	0	0	0	0%
**Anosmia**	0	0	0	0	0	0	0	0	0	0	0	0%
**Coryza**	0	0	0	1	0	0	0	0	0	0	0	9.1%
**Cough**	0	1	1	1	0	0	0	0	0	0	0	27.3%
**Diarrhea**	0	0	0	0	0	0	0	0	0	0	1	9.1%
**Dyspnea**	0	0	1	0	0	0	0	1	1	1	0	36.4%
**Fatigue**	0	0	0	1	0	0	0	1	0	0	0	18.2%
**Fever**	0	1	0	0	1	0	1	0	1	1	1	54.5%
**Headache**	1	0	0	0	1	0	0	0	1	1	0	36.4%
**Respiratory distress**	0	0	0	0	0	0	0	0	1	0	0	9.1%
**Sore throat**	0	0	0	0	0	0	0	0	0	0	0	0%
**Vomit**	0	0	0	0	0	0	0	0	0	0	0	0%
**Myalgia**	0	0	0	1	0	0	0	1	1	1	0	36.4%
**Others**	Chills	Pain in chest						Joint pain			Odynophagia	

* Strain P.1.1
** Re-infection with P.1

References:

S1: Fabiani M, Margiotti K, Viola A, et al. Mild Symptomatic SARS-CoV-2 P.1 (B.1.1.28) Infection in a Fully Vaccinated 83-Year-Old Man. Pathogens. 2021;10(5):614. doi: 10.3390/pathogens10050614

S2: Campoy PJS, Buenestado-Serrano S, Pérez-Lago L, et al. First importations of SARS-CoV-2 P.1 and P.2 variants from Brazil to Spain and early community transmission. Enferm Infecc Microbiol Clin. 2021. doi: 10.1016/j.eimc.2021.05.008

S3: da Silva JC, Félix VB, Leão SABF, et al. New Brazilian variant of the SARS-CoV-2 (P1/Gamma) of COVID-19 in Alagoas state. Braz J Infect Dis. 2021;25(3):101588. doi: 10.1016/j.bjid.2021.101588

S4: Angeletti S, Giovanetti M, Fogolari M, et al. Detection of a SARS-CoV-2 P.1.1 variant lacking N501Y in a vaccinated health care worker in Italy. J Infect. 2021;S0163-4453(21)00325-X. doi: 10.1016/j.jinf.2021.06.026

S5: Maggi F, Novazzi F, Genoni A, et al. Imported SARS-CoV-2 Variant P.1 in Traveler Returning from Brazil to Italy. Emerg Infect Dis. 2021;27(4):1249-1251. doi: 10.3201/eid2704.210183

S6: Hirotsu Y, Omata M. Discovery of a SARS-CoV-2 variant from the P.1 lineage harboring K417T/E484K/N501Y mutations in Kofu, Japan. J Infect. 2021;82(6):276-316. doi: 10.1016/j.jinf.2021.03.013

S7: Romano CM, Felix AC, Paula AV, et al. SARS-CoV-2 reinfection caused by the P.1 lineage in Araraquara city, Sao Paulo State, Brazil. Rev Inst Med Trop Sao Paulo. 2021;63:e36. doi: 10.1590/S1678-9946202163036

S8: de Siqueira IC, Camelier AA, Maciel EAP, et al. Early detection of P.1 variant of SARS-CoV-2 in a cluster of cases in Salvador, Brazil. Int J Infect Dis. 2021;108:252-255. doi: 10.1016/j.ijid.2021.05.010

Supplementary material (S.2)

Data analyzed

As of 2nd August 2021, the raw data were downloaded in the form of two csv files with links available in the bottom of the official web page of the case dashboard, "Amazonas COVID-19 Panel" (*Painel COVID-19 Amazonas*), at http://www.saude.am.gov.br/painel/corona/. The two data files contained all cases confirmed in Amazonas in 2020 and 2021. For analysis, the data of both files were combined into one csv file then removal of cases not meeting the inclusion criteria was performed as described in Supplementary material (S.2) below.

The data shown in the "Amazonas COVID-19 Panel" were collected by the Amazonas Health Surveillance Foundation (*Fundação de Vigilância em Saúde do Amazonas*) from the health information systems of municipal health departments (*Secretarias Municipais de Saúde*). The Amazonas Health Surveillance Foundation is an arm of the state government of Amazonas responsible for the public health of Amazonas, including the management of the health information systems.

The database contains all COVID-19 cases in Amazonas based on the information from three sources, namely e-SUS Notifica, the Influenza Epidemiological Surveillance Information System (*Sistema de Informação da Vigilância Epidemiológica da Gripe*, SIVEP-Gripe) and the Laboratory Environment Manager (*Gerenciador de Ambiente Laboratorial*, GAL). The e-SUS Notifica is generally for the reporting of mild COVID-19 cases whereas SIVEP-Gripe is for hospitalized cases. The GAL is a system for the management of the National Network of Epidemiological Surveillance Laboratories (*Rede Nacional de Laboratórios de Vigilância Epidemiológica*), part of the SUS (*Sistema Único de Saúde*), Brazil's government-funded public health care system. The GAL manages and monitors the steps to carry out the tests and to obtain analytical and epidemiologic reports in the network of public health laboratories that consists of government laboratories and accredited private laboratories.

Consequently, the database contains all confirmed cases in Amazonas, irrespective of disease severity, meaning that those not admitted to the hospital and those in home isolation were also included. This is different from the previously reported nationwide database SIVEP-Gripe (*Sistema de Informação de Vigilância Epidemiológica da Gripe*) that only considers hospitalized cases and death without hospital admission.

Supplementary material (S.3)

Grouping of municipalities according to the Brazilian Institute of Geography and Statistics (*Instituto Brasileiro de Geografia e Estatística, IBGE*)

Municipality	Intermediate geographic region
Alvaraes	Tefé
Anama	Manaus
Anori	Manaus
Apui	Lábrea
Atalaia Do Norte	Tefé
Autazes	Manaus
Barcelos	Manaus
Barreirinha	Parintins
Benjamin Constant	Tefé
Beruri	Manaus
Boa Vista Do Ramos	Parintins
Boca Do Acre	Lábrea
Borba	Manaus
Canutama	Lábrea
Carauari	Tefé
Careiro	Manaus
Careiro Da Varzea	Manaus
Coari	Manaus
Codajas	Manaus
Eirunepe	Tefé
Envira	Tefé
Fonte Boa	Tefé
Humaita	Lábrea
Ipixuna	Tefé
Iranduba	Manaus
Itacoatiara	Parintins
Jurua	Tefé
Jutai	Tefé

Municipality	Intermediate geographic region
Labrea	Lábrea
Manacapuru	Manaus
Manaquiri	Manaus
Manaus	Manaus
Manicore	Lábrea
Maraa	Tefé
Maues	Parintins
Nhamunda	Parintins
Nova Olinda Do Norte	Manaus
Novo Airao	Manaus
Novo Aripuana	Lábrea
Parintins	Parintins
Presidente Figueiredo	Manaus
Rio Preto Da Eva	Manaus
Santa Isabel Do Rio Negro	Manaus
Santo Antonio Do Ica	Tefé
Sao Gabriel Da Cachoeira	Manaus
Sao Paulo De Olivenca	Tefé
Sao Sebastiao Do Uatuma	Parintins
Silves	Parintins
Tabatinga	Tefé
Tapaua	Lábrea
Tefe	Tefé
Tonantins	Tefé
Uarini	Tefé
Urucara	Parintins
Urucurituba	Parintins

Supplementary material (S.4)

Further breakdown of the cases identified in the database

The below flow chart shows the further breakdown of data identified in the database. A total of 404,262 COVID-19 cases were identified in the two data files, as described in Supplementary material (S.1). Sever rounds of case removal took place. Cases not diagnosed with PCR tests, patients aged 18 years or above, asymptomatic cases, cases confirmed outside of the study time periods were not included in statistical analyses.

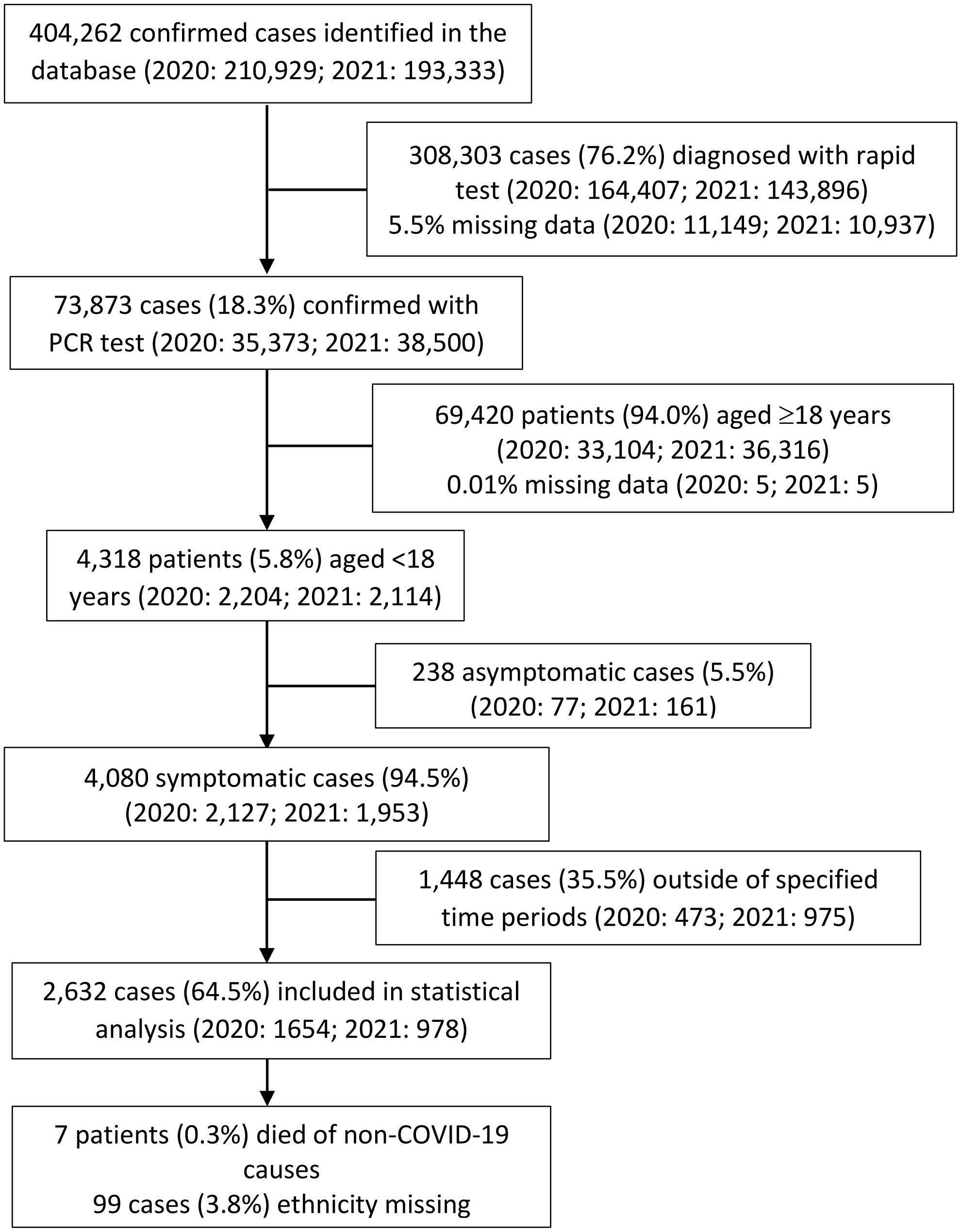

Supplementary material (S.5)

Univariate analysis

	Univariate OR (95% CI)
Age	1.025 (1.012,1.039)
Male	0.797 (0.68,0.934)
Age groups	
Infants	1.044 (0.842,1.294)
Young children	0.743 (0.605,0.911)
Children	0.729 (0.605,0.879)
Adolescents	1.509 (1.284,1.773)
Fatality	
All	0.526 (0.266,1.041)
Non-infants	0.379 (0.166,0.865)
Infants	1.647 (0.406,6.681)
Young children	0.21 (0.027,1.656)
Children	0.472 (0.101,2.204)
Adolescents	0.436 (0.14,1.36)
Ethnicity	
Latino	1.443 (1.145,1.819)
Asian	0.458 (0.209,1.003)
Caucasian	1.147 (0.859,1.532)
Indigenous	0.526 (0.341,0.81)
Location	
Manaus	0.885 (0.734,1.067)
Parintins	1.768 (1.103,2.835)
Tefé	0.774 (0.599,0.999)
Lábrea	1.468 (1.117,1.93)
Signs and symptoms	
Ageusia	4.868 (3.551,6.672)
Anosmia	1.477 (1.171,1.862)
Coryza	8.776 (6.543,11.772)
Cough	0.833 (0.706,0.981)
Diarrhea	0.316 (0.21,0.474)
Dyspnea	0.734 (0.618,0.873)
Fatigue	2.388 (1.56,3.657)
Fever	0.842 (0.706,1.004)
Headache	12.031 (9.454,15.311)
Respiratory distress	0.571 (0.454,0.719)
Sore throat	1.492 (1.263,1.763)
Vomit	0.413 (0.284,0.601)

Others	0.812 (0.653,1.011)
Comorbidities	
No comorbidities	1.619 (1.147,2.287)
1 comorbidity	0.649 (0.433,0.972)
2 comorbidities	0.755 (0.391,1.459)
≥ 3 comorbidities	0 (0,Inf)
Heart disease	1.271 (0.534,3.027)
Hematologic disease	0 (0,Inf)
Neurological disease	0.504 (0.202,1.26)
Hepatic disease	0 (0,Inf)
Renal disease	0.724 (0.187,2.806)
Immunodeficiency	0.631 (0.292,1.363)
Down syndrome	1.057 (0.345,3.241)
Obesity	2.26 (0.505,10.119)
Diabetes	0.211 (0.026,1.686)
Others	0.493 (0.307,0.791)

Supplementary material (S.6)

Receiver operating characteristic (ROC) curve

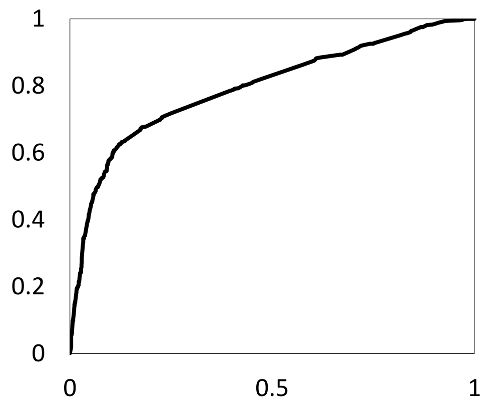

Supplementary material (S.7)

Sensitivity analysis

	Multivariate ORs	p-value
Male	0.723 (0.563,0.927)	0.011
Children	0.752 (0.583,0.97)	0.028
Black/brown	1.923 (1.331,2.778)	<0.001
Ageusia	2.142 (1.361,3.373)	0.001
Coryza	4.243 (2.716,6.629)	<0.001
Diarrhea	0.345 (0.169,0.705)	0.003
Fatigue	3.549 (1.868,6.743)	<0.001
Fever	0.69 (0.526,0.904)	0.007
Headache	10.285 (7.472,14.155)	<0.001
Other symptoms	0.659 (0.47,0.924)	0.016
Hematologic disease	0 (0,Inf)	0.974
Parintins	3.172 (1.556,6.465)	0.001
Lábrea	1.781 (1.201,2.64)	0.004

Supplementary material (S.8)

(a) Monthly prevalence rate of each sign and symptom.

	Ageusia	Anosmia	Coryza	Cough	Diarrhea	Dyspnea	Fatigue	Fever	Headache	Respiratory discomfort	Sore throat	Vomit	Others
Mar-20	0.00%	0.00%	0.00%	90.00%	10.00%	50.00%	0.00%	80.00%	0.00%	40.00%	60.00%	0.00%	20.00%
Apr-20	0.00%	4.97%	0.00%	80.11%	2.76%	38.67%	1.10%	80.66%	0.55%	18.78%	40.88%	3.87%	37.02%
May-20	1.13%	14.69%	0.00%	65.25%	9.89%	39.55%	0.28%	85.03%	0.85%	22.32%	25.71%	7.34%	16.10%
Jun-20	0.40%	12.90%	0.00%	50.81%	17.34%	31.05%	1.21%	64.52%	0.00%	20.97%	17.74%	14.11%	6.85%
Jul-20	1.65%	12.35%	1.65%	70.37%	6.17%	38.68%	0.82%	74.90%	3.29%	19.34%	35.80%	7.00%	20.16%
Aug-20	8.24%	11.76%	1.18%	72.35%	8.24%	36.47%	7.65%	82.94%	4.12%	21.18%	30.59%	8.82%	17.06%
Sep-20	6.03%	6.03%	12.93%	62.07%	9.91%	27.16%	5.60%	65.52%	15.52%	12.50%	26.72%	12.50%	13.79%
Oct-20	9.72%	11.57%	11.57%	66.67%	4.63%	27.78%	1.85%	63.43%	19.44%	14.81%	31.94%	5.09%	15.28%
Nov-20	17.39%	16.30%	10.33%	71.20%	7.61%	32.61%	10.87%	67.39%	20.65%	21.20%	29.89%	13.04%	12.50%
Dec-20	14.88%	19.38%	9.69%	67.13%	9.34%	35.64%	7.27%	73.36%	15.57%	20.76%	28.37%	8.65%	11.07%
Jan-21	9.26%	14.35%	18.67%	65.28%	4.32%	32.72%	4.94%	68.98%	34.41%	14.81%	39.97%	4.48%	16.36%
Feb-21	13.76%	15.60%	20.18%	63.61%	8.26%	28.75%	6.73%	71.25%	34.25%	13.76%	37.31%	5.81%	10.09%
Mar-21	14.29%	16.45%	18.18%	61.04%	6.49%	30.74%	9.09%	66.67%	39.83%	20.78%	35.93%	6.49%	15.15%
Apr-21	16.03%	14.74%	21.15%	64.74%	2.56%	35.90%	4.49%	60.26%	35.90%	16.67%	32.69%	7.05%	10.90%
May-21	13.36%	13.79%	29.74%	58.62%	2.59%	23.28%	3.02%	68.97%	40.52%	7.33%	35.34%	2.16%	17.67%
Jun-21	16.52%	16.07%	24.55%	62.05%	0.45%	25.45%	5.80%	79.91%	47.32%	9.38%	39.73%	1.34%	13.84%
Jul-21	15.56%	16.30%	34.81%	65.19%	2.22%	25.93%	2.96%	77.78%	52.59%	2.22%	51.11%	1.48%	13.33%

(b) Correlation analysis between signs/symptoms and linealprevalence

The below correlations were calculated based on the linealprevalence reported in Figure 1 and the monthly prevalence rates in (a) above.

	Ageusia	Anosmia	Coryza	Cough	Diarrhea	Dyspnea	Fatigue	Fever	Headache	Respiratory discomfort	Sore throat	Vomit	Others
B.1 & B.1.1	-0.049	-0.316	-0.403	0.463	0.280	0.273	0.292	-0.026	-0.420	0.423	-0.063	0.384	-0.012
B.1.1.28	-0.613	-0.363	-0.686	0.016	0.525	0.146	-0.288	-0.111	-0.740	0.216	-0.523	0.616	0.156
B.1.1.33	-0.770	-0.489	-0.803	0.281	0.540	0.531	-0.376	0.464	-0.831	0.494	-0.226	0.306	0.243
Delta (B.1.617.2)	0.284	0.173	0.273	-0.143	-0.384	-0.290	0.115	0.255	0.364	-0.257	0.117	-0.317	-0.074
P.2	0.342	0.324	0.066	0.016	-0.022	-0.122	0.386	-0.342	0.114	-0.014	-0.115	0.168	-0.210
P.1	0.718	0.527	0.881	-0.410	-0.575	-0.532	0.297	-0.200	0.914	-0.567	0.272	-0.470	-0.282
Others	-0.691	-0.670	-0.739	0.730	0.338	0.784	-0.460	0.415	-0.765	0.719	0.228	0.021	0.532
